# Personalized Medicine: New Perspectives for the Diagnosis and the Treatment of Renal Diseases

**DOI:** 10.3390/ijms18061248

**Published:** 2017-06-10

**Authors:** Anna Gluba-Brzózka, Beata Franczyk, Robert Olszewski, Maciej Banach, Jacek Rysz

**Affiliations:** 1 Department of Nephrology, Hypertension and Family Medicine, WAM Teaching Hospital, Lodz 90-549, Poland; 2 Department of Nephrology, Hypertension and Family Medicine, Medical University of Lodz, Lodz 90-549, Poland; bfranczyk-skora@wp.pl (B.F.); jacek.rysz@umed.lodz.pl (J.R.); 3 Department of Ultrasound, Institute of Fundamental Technological Research, Polish Academy of Sciences (IPPT PAN), Warsaw 02-106, Poland; robert.olszewski@me.com; 4 Department of Hypertension, Medical University of Lodz, Lodz 90-549, Poland; maciej.banach@umed.lodz.pl

**Keywords:** renal diseases, personalized medicine, treatment, diagnosis, biomarkers

## Abstract

The prevalence of renal diseases is rising and reaching 5–15% of the adult population. Renal damage is associated with disturbances of body homeostasis and the loss of equilibrium between exogenous and endogenous elements including drugs and metabolites. Studies indicate that renal diseases are influenced not only by environmental but also by genetic factors. In some cases the disease is caused by mutation in a single gene and at that time severity depends on the presence of one or two mutated alleles. In other cases, renal disease is associated with the presence of alteration within a gene or genes, but environmental factors are also necessary for the development of disease. Therefore, it seems that the analysis of genetic aspects should be a natural component of clinical and experimental studies. The goal of personalized medicine is to determine the right drug, for the right patient, at the right time. Whole-genome examinations may help to change the approach to the disease and the patient resulting in the creation of “personalized medicine” with new diagnostic and treatment strategies designed on the basis of genetic background of each individual. The identification of high-risk patients in pharmacogenomics analyses will help to avoid many unwarranted side effects while optimizing treatment efficacy for individual patients. Personalized therapies for kidney diseases are still at the preliminary stage mainly due to high costs of such analyses and the complex nature of human genome. This review will focus on several areas of interest: renal disease pathogenesis, diagnosis, treatment, rate of progression and the prediction of prognosis.

## 1. Introduction

The prevalence of chronic kidney diseases is rising and reaching 5–15% of the adult population [1,2]. The kidney is an organ, which acts as a blood filter, it participates in the regulation of electrolytes levels and acid-base balance. Moreover, kidneys control blood pressure, modulate water imbalance, they are engaged in the reabsorption of water, glucose and amino acids and excrete urea and ammonium. Therefore, kidney damage is associated with disturbances of body homeostasis as well as loss of equilibrium between exogenous and endogenous elements including drugs and metabolites [3].

The goal of personalized medicine is to determine the right drug, for the right patient, at the right time. Moreover, it is focused on the tailoring and timing of preventive and therapeutic measures based on biological information and biomarkers on genetics, proteomics as well as metabolomics and the level of molecular disease pathways [4,5]. Improvements in molecular biology techniques and associated bioinformatics tools have led to the introduction of cost-effective genotyping approaches, such as high-density microarrays (allowing for the analysis of up to 5 million single-nucleotide polymorphisms; SNPs) and next-generation sequencing (NSG) enabling the examination of individual’s entire genome [3]. Moreover, fluorescence resonance energy transfer (FRET) based assays, locked nucleic acid (LNA) technology, comparative genomic hybridization (CGH), fluorescent in situ hybridization, functional MRI with nanoparticle contrast, FDG-PET and many other techniques are also used in the analysis of genome and molecular interactions. Human genome sequencing (completed in 2000 [6]) as well as the Human Genome Project (completed by 2003) which identified 22,000–23,000 genes in the human genome [7] were first large projects which laid the foundations of personalized medicine [8]. However, the knowledge of genome composition provided little understanding of mechanisms occurring in our body and interactions with environment. Therefore, studies taking into account both genetic and environmental factors were necessary. Epigenetics focuses on heritable changes in gene expression pattern which is mediated by mechanisms other than variations in the primary nucleotide sequence of genes [9]. Epigenetic changes which are responsible for the regulation of gene expression, silencing of the activity of transposable elements and the determination of gene dosage in the case of chromosome X inactivation and genomic imprinting are associated with mechanisms such as DNA methylation, histone acetylation/deacetylation, histone methylation and RNA interference [9,10]. Also the role of non-coding RNAs and antisense transcripts in the genome as well as their functional consequences are of high interest now [11]. It has been revealed that also protein product of one gene may influence the expression of another gene or it can affect the functionality of other protein (e.g., via phosphorylation or cleavage) [12]. Moreover, protein encoded by two different genes may form a complex which function depends on both. Therefore, the detection and characterization of such molecular interactions is important in the understanding of mechanisms occurring in our bodies and determining health condition. So far, genome-scale comparisons of protein-protein network organization remain a challenge, despite the introduction of new technology, named chromatin immunoprecipitation (ChIP)-chip or ChIP-Seq [12]. New fields of medical sciences, including transcriptomic, proteomic and metabolomic provide much more valuable information than genomic sequences alone. The combination of genomic information with the aforementioned omics should in the future provide real-time information of a person’s physiological status. Among the most important projects aiming at the unravelling of human genome there are: the aforementioned Human Genome Project (2003) [7], Phase I HapMap project completed in 2005 (in which more than one million single nucleotide polymorphisms for which accurate and complete genotypes have been obtained in 269 DNA samples from four populations) [13], Personal Genome Project initiated by George M. Church of Harvard University in 2005 (focusing on individualizing disease risk factors, biological characteristics, and personal ancestries) [14], National Institutes of Health co-sponsored Genome-Wide Association Studies (GWAS) (focused on the scanning of markers across the complete sets of DNA, or genomes, of many people to find genetic variations associated with a particular disease) [15], ongoing encyclopedia of DNA Elements (aiming at the identification of all functional elements preliminary in the human genome sequence) [16] as well as 1000 Genomes Project aiming at deep characterization of human genome sequence variation as a foundation for investigating the relationship between genotype and phenotype [17,18,19]. Apart from projects covering the whole genome, there are also projects concentrated on a selected type of disease, e.g., the Cancer Research UK 2005 (the first and largest ever genetic research into breast cancer aiming at the understanding of the genetic basis of the disease in the area of prevention, early detection, and treatment) [20], National Institute of Health founded research into the understanding of the genetic basis of coronary heart disease, stroke, and breast cancer in relation to postmenopausal hormone therapy in 161,808 women between the ages of 50 and 79 [21]. 

High throughput sequencing of major tumors has improved the understanding of molecular mechanisms of tumor transformation and progression [22] and led to the development of several oncogenes-targeting therapies, including the c-KIT inhibitor Imatinib, the anti-HER2 agent Trastuzumab and the ALK inhibitor Crizotinib for the treatment of chronic myeloid leukemia (CML), non-small cell lung cancer (NSCLC) and breast cancer [23]. Trastuzumab was one of the earliest personalized therapy discoveries which addition to standard chemotherapy significantly improved survival for patients with human epidermal growth factor receptor 2 positive (HER2+) disease [24].

However, according to available data, personalized therapies for kidney diseases are still at the preliminary stage. This review will focus on several areas of interest: renal disease pathogenesis, diagnosis, treatment, rate of progression and the prediction of prognosis. 

## 2. Renal Disease Diagnosis or Early Detection

Owing to the advances in molecular biology techniques (i.e., NGS, qRT-PCR) it is now possible to identify key molecules and pathways engaged in the pathogenesis of renal diseases, including the development of renal fibrosis or kidney disease progression and renal cell function [25,26,27]. There is an increasing interest in the role of micro ribonucleic acids (miRNAs) in the pathogenesis of renal diseases [23,28]. miRNAs, which are small non-coding RNAs implicated in post-transcriptional silencing, can regulate multiple transcripts and therefore influence signalling pathways [29]. For the time being, several miRNAs have been involved in various renal diseases, including diabetic nephropathy, glomerulonephritis and polycystic kidney disease [30,31,32].

Genetic variants are responsible for many genetic renal diseases, such as autosomal dominant polycystic kidney disease (ADPKD), congenital nephrotic syndrome, congenital abnormities of the kidney, etc. Conventional diagnosis methods are time-consuming and often lacking sensitivity and specificity. Renal biomarkers currently used in CKD diagnosis and the assessment of its course, such as estimated glomerular filtration rate (eGFR), albuminuria, creatinine, urea, uric acid, cystatin C are lacking appropriate sensitivity and specificity for early stage CKD patients, which leads to the delayed intervention [33]. Nowadays, sequencing and microarray techniques are used in biomarker-related research. Urinary microRNA (miRNAs) has been demonstrated to be useful in early detection and predicting renal impairment progression. 

Molecular diagnostics of autosomal dominant polycystic kidney disease (ADPKD), which is the most common inherited kidney disease, relies on mutation screening of PKD1 (16p13.3; OMIM ID: 601313) and PKD2 (4q21; OMIM ID: 173910) [34]. Mutations in PKD1 and PKD2 genes have been found in over 90% of patients with ADPKD. 8% to 10% of patients do not have any of them, and some studies suggest the presence of mutation in a third gene—GANAB. However, this was shown to account for only a small number of these PKD1 and PKD2 mutation-negative patients [35]. ADPKD is characterized by the development and enlargement of cysts within the kidneys and other organs, which finally lead to end-stage renal disease (ESRD). In children, glomerular hyperfiltration, microalbuminuria, loss of urinary concentration and loss of the normal diurnal blood pressure rhythm were also observed [36,37]. The phenotype of ADPKD is diverse and considerably depends on the affected gene. Milder symptoms and prolonged time to progression to ESRD is observed in patients with PDK2. The diagnosis of ADPKD is based on renal imaging, including ultrasonography, computed tomography, or magnetic nuclear resonance; however, in some situations molecular diagnostics is necessary. Due to allelic heterogeneity and the presence of six highly homologous sequences of *PKD1*, its sequencing required long-range amplifications. However, recently Trujillano et al. [34] described new method of the detection of ADPKD pathogenic mutations—in-solution hybridization enrichment coupled to NGS. According to authors, it is cost- and time-effective, and meets the sensitivity and specificity criteria required for genetic diagnostics. In the past ADPKD was considered an untreatable disease, however now, due to accurate diagnostics tools, the prognostic scoring and the development of new drugs is possible. Constant search for new therapeutics and enhanced interest in patient-centered options are likely to alter the long-term management of ADPKD for patients and their families [37]. 

Currently, in clinical practice, renal damage is usually diagnosed on the basis of proteinuria/albuminuria in urinalysis or quantitative measurement of creatinine concentration and the calculation of eGFR. However, these methods are non-specific and do not always provide accurate information on the risk of progression. A good biomarker of CKD should accurately predict an individual’s risk of CKD progression or developing hard renal end points (e.g., ESKD or death), identify additional risk factors for CKD, indicate and quantify a pathological process within the kidney, which may or may not be modifiable and finally it should act as an indicator of treatment response [33]. Therefore, there is a need for alternative biomarkers of renal damage enabling early and more accurate disease assessment [38]. 

Early detection of high risk CKD patients combined with the introduction of secondary prevention focused on blood pressure, glycaemic and lipid control should improve the prognosis of patients with CKD. Treatment of high blood pressure is of supreme importance for preventing and delaying the progression of CKD, while the reduction of albuminuria hampers the progression rate of renal function loss in these patients. Albuminuria is not only a symptom of renal impairment but it can also promote renal damage itself. A low protein diet [39], RAAS blockade, non-steroidal anti-inflammatory drugs [40] and corticosteroids [41] are used to lower blood pressure, decrease albuminuria, provide renoprotection and slow progression to ESRD. However, the effects of these therapies or strategies are not necessarily satisfactory [42].

Acute kidney injury (AKI) is associated with high mortality, and therefore its early diagnosis is necessary to facilitate early intervention aimed at limiting related morbidity and mortality [43]. Currently, serum creatinine is the standard marker in the detection of AKI, however, it is not a perfect early indicator. In the study of Herget-Rosenthal et al. [43] cystatin C was suggested to be a better marker of AKI with sensitivity and specificity values of 0.82 and 0.97 in a group of patients at high AKI risk. According to R-criteria, cystatin C detected AKI with a sensitivity of 55% and 82% on the two days before the R-criteria was fulfilled by creatinine, respectively. Also according to I- and F-criteria, detection made on the basis of cystatin was two days prior to creatinine. Moreover, it was useful in the prediction of renal replacement therapy in the further course of AKI [43]. 

Glomerular diseases involves wide group of histologically defined primary glomerulopathies (e.g., focal segmental glomerulosclerosis (FSGS), membranous glomerulonephritis (MGN), minimal change disease (MCD), and IgA nephropathy (IgAN)) and secondary glomerulopathies due to diabetes mellitus (diabetic nephropathy), systemic autoimmune disorders (e.g., lupus erythematosus and haemolytic-uremic syndrome), or chronic viral infection (e.g., hepatitis and HIV) [38]. Good et al. [38] performed an analysis of samples from 3600 individuals using capillary electrophoresis coupled to MS to define urinary peptide biomarkers for chronic kidney diseases in order to diagnose these diseases with high accuracy. Urine is a rich source of biomarkers for a wide range of diseases, which can be used in non-invasive diagnosis. Moreover, this type of biological material is quite stable. In Good et al. [38] study, the application of chronic kidney disease-specific biomarker resulted in 85.5% sensitivity and 100% specificity in distinguishing healthy subjects from individuals with biopsy-proven kidney disease. Various collagens, blood proteins (e.g., α1-antitrypsin, serum albumin, haemoglobin α chain, and fibrinogen α chain), kidney-specific proteins (e.g., uromodulin, sodium/potassium-transporting ATPase γ chain, and membrane-associated progesterone receptor component 1) and fragments of different secreted proteins have been identified as markers for the diagnosis of CKD. Advanced chronic kidney disease, irrespective of its aetiology, is characterized by tubular atrophy, interstitial fibrosis, and glomerulosclerosis. The decreased activity of matrix metalloproteases was suggested to be responsible for the accumulation of proteins in the extracellular matrix and collagens that mediate the fibrotic kidney [44]. Also α_1_-Antitrypsin and its fragments were demonstrated to be up-regulated in several types of CKD [45]. Moreover, reduced urinary levels of these collagen- and uromodulin-derived peptide fragments were suggested to serve as indicators of (patho)physiological changes in CKD [38].

Neutrophil Gelatinase-Associated Lipocalin (NGAL) is rapidly released by renal tubules in response to injury. Studies of both paediatric and adult populations indicated acute rise in urinary NGAL (determining evolving AKI) within 2 to 8 h of cardiac surgery [46,47]. In normal conditions, NGAL is involved in the formation of renal epithelium. 

In Mishra et al. [48] study, neutrophil gelatinase-associated lipocalin (NGAL), also known as lipocalin-2 (LCN2), was found to be a promising biomarker for the early detection of acute renal failure. In their study, the observed sensitivity and specificity for urine NGAL concentrations reached values of 1.00 and 0.98, respectively [49]. 

However, a prospective observational study of 426 adults who underwent cardiac surgery demonstrated that the relationship between postoperative urinary NGAL and AKI varied with baseline renal function. Positive relationship was observed in this study only in patients with preoperative eGFR ≥ 60 mL/min. Authors suggested that the ability of urinary NGAL to provide early and accurate identification of developing AKI was optimal in patients with normal baseline renal function (eGFR 90 to 120 mL/min) [50].

The aetiology and pathogenesis of focal segmental glomerulosclerosis (FSGS), which is a common form of idiopathic nephrotic syndrome characterized by proteinuria and focal glomerular sclerosis lesions, are still unknown. FSGS is an important cause of end-stage renal disease. The development of novel methods of molecular biology is used to analyse these issues. A better understanding of molecular mechanisms leading to the development and progression of this disease should simplify the design of more effective FSGS-specific treatment [51]. Unique renal cortical RNA expression enables the differentiation of focal segmental glomerulosclerosis (FSGS) with nephrotic-range proteinuria or renal insufficiency from FSGC without the accompanying symptoms in paediatric patients [52]. Detailed gene-expression profiling of isolated glomeruli from patients with biopsy-proven idiopathic classic FSGS, collapsing FSGS, MCD, and normal controls demonstrated that podocyte injury played a central role in the pathogenesis of FSGS. Hodgin et al. [51] observed differential expression of several transcripts of proteins involved in podocyte slit diaphragm structure and function. Nephrin, podocin and synaptopodin expression was shown to be decreased in disease conditions. Differential expression of *FAT1* (down 2-fold), *GRB2* (up 2.1-fold), *MAGI-2* (down 4.2-fold), and *TJP1* (down 1.8-fold, also called *ZO1*) genes was also seen. Moreover, they observed significantly increased (9.8-fold) expression of *PAX2*, which is a marker for the immature podocyte, as well as decreased expression of several markers for the mature podocyte, including *CALLA* (2.7-fold, also known as *MME* or *CD10*), *CR1* (2.2-fold), podocalyxin (2.3-fold), and synaptopodin (3.3-fold) [51]. This study provided new data concerning the molecular pathogenesis of glomerular injury and structural degeneration in FSGS in which processes of development, differentiation and morphogenesis, cell motility and migration, cytoskeleton organization, and signal transduction were overrepresented. It seems that also the reactivation of developmental programs plays an important role in the pathogenesis of FSGS [51]. Some studies hypothesized that suPAR was a circulating factor causing FSGS. The study of Wei et al. [53] reported that sera from patients with post-transplant FSGS recurrence activated podocyte αvβ3-integrin. Moreover, in uPAR-null mice dose-dependent proteinuria was observed following the injection of a chimeric full-domain murine suPAR linked to a human IgG1-Fc. A fragment of suPAR, produced by a plasmid and containing domains 1 and 2 in wild-type mice resulted in proteinuria and early FSGS lesions in mice. Maas et al. [54] study demonstrated an inverse correlation between suPAR and eGFR in a small group of patients with primary and secondary FSGS, and MCNS, however, serum suPAR concentration did not distinguish between respective patient groups [55]. Some other studies failed to validate the role of intact suPAR and distinguish FSGS and other glomerular diseases in patients with elevated suPAR [56,57].

Sharma et al. [57] identified cardiotrophin-like cytokine factor 1 (CLCF1) in the plasma from patients with recurrent FSGS. The analysis of effect of CLCF1 on isolated rat glomeruli using an in vitro assay of albumin permeability (P(alb)) demonstrated that CLCF1 (0.05–100 ng/mL) increased P(alb) and caused maximal effect at 5–10 ng/mL (*p* < 0.001). The elevation in Palb was equivalent to the effect of FSGS serum. Moreover, anti-CLCF1 monoclonal antibody was shown to block CLCF1-induced increase in P(alb) and significantly reduced the effect of FSGS serum (*p* < 0.001). Additionally, CLCF1 upregulated phosphorylation of signal transducer and activator of transcription 3 (STAT3) (Tyr705) in glomeruli, Janus kinase 2 (JAK2) inhibitor BMS-1119543 or STAT3 inhibitor Stattic significantly blocked the effect of CLCF1 or FSGS serum on P(alb) (*p* < 0.001). It seems that CLCF1 in monomeric form increases P(alb), while heterodimer CLCF1–CRLF1 may protect the glomerular filtration barrier. Authors of this paper hypothesized that albuminuria in FSGS was associated with qualitative or quantitative changes in the CLCF1–CRLF1 complex, and that JAK2 or STAT3 inhibitors might become a novel therapeutic agents to treat FSGS [57].

Currently, the diagnosis of membranous glomerulopathies is made on the basis of the presence of granular deposits of IgG in renal biopsy together with glomerular basement membranes on immunofluorescence and subepithelial deposits visible in electron microscopy [58]. This disease can be divided into primary condition with unknown etiology and secondary when one of numerous associated conditions is identified. Owing to the identification of PLA2R as the antigenic target in ~70% of primary membranous glomerulopathies, the diagnosis of a specific subtype of membranous glomerulopathies on the basis of underlying mechanism will be possible. Moreover, serologic testing for PLA2R antibodies could be used in non-invasive disease monitoring [59]. The ability to classify more of membranous glomerulopathies cases has been recently widened by the identification of THSD7A serving as a second antigenic target of antibodies [58].

## 3. Susceptibility and Rate of Disease Progression

Biomarkers can be used to predict impending disease activity in order to implement early treatment to improve outcomes, decrease chronic renal injury and minimize the required treatment [3]. Moreover, biomarkers could help to predict response to therapy and therefore to adjust the treatment regimen to individual patient. Due to the fact that they mirror disease severity, biomarkers can be utilized to tailor therapy intensity. However, since the applications of new markers are limited they probably will not replace current practice. Otu et al. [60] utilized urinary protein profiling to predict the development of diabetic nephropathy (DN) in patients with type 2 diabetes and with no signs of renal disease, followed for 10 years. On the basis of surface-enhanced laser desorption/ionization time-of-flight mass spectrometry (SELDI-TOF MS) of urine samples it was possible to predict with a sensitivity of 71%, a specificity of 76%, and a positive predictive value of 74%, which patients were destined to develop nephropathy. However, for the time being, none of the 12 proteins involved in specific DN signature has yet been identified. Merchant et al. [61] analysed urine samples (using liquid chromatography–matrix-assisted laser desorption/ionization TOF MS) collected from patients with type 1 diabetes, microalbuminuria but normal renal function in order to identify biomarkers that would indicate patients who would progress quickly to chronic kidney disease and those who would not. They found that three peptides derived from collagen types IV and V and tenascin-X were decreased in the urine of patients with deteriorating renal function, while fragments of inositol pentakisphosphate 2-kinase, zona occludens 3, and FAT tumor suppressor 2 were increased in this group. 

The first genome-wide association scan for renal traits performed as a part of Framingham Heart Study (FHS), which included 70,987 single nucleotide polymorphisms (SNPs) and 1010 FHS participants provided a list of SNPs with strong association with renal disease [62]. Sixteen of them (the~most promising) have been then analysed in 15,747 participants of the Atherosclerosis in Communities (ARIC) study. This analysis demonstrated that intronic SNP (rs6495446) in methenyltetrahydrofolate synthetase (MTHFS) gene was significantly associated with CKD among white ARIC participants indicating the possible involvement of this gene in disease development. Another linkage analysis performed on 848 participants from 26 Mexican American families (San Antonio Family Heart Study; SAFHS) found linkage on chromosomal regions 2p25, 20q12 and 9q21 for several parameters of renal function (S-creatinine, creatinine clearance and estimated glomerular filtration rate) [63]. Quantitative trait loci (QTLs) for SrCr and eGFR were identified in this study in the same chromosomal region (9q21). In the same region, genes coding cathepsin L (CTSL), which is implicated in the renal tubular response to proteinuria and myocardial ischaemia [64], proprotein convertase 5 (PC5), which is colocalized with α V with inflammation-related phenotypes [64] as well as transient receptor potential cation channel subfamily M member 3 (TRPM3) which plays a role in volume-regulated activity and renal calcium homeostasis [32] are also localized. Moreover, in the case of creatinine clearance (CrCl) evidences of linkage on chromosome 2 (2p25) has been found in SAFHS study [63].

Whole genome mapping by admixture linkage disequilibrium (MALD) has been performed by Kao et al. [65] in order to look for ESRD genes that are present at higher frequency in African than in European Americans. In their study, the scan of 1372 ESRD cases and 806 controls enabled the identification of a locus on chromosome 22q12 which showed a significant excess of African ancestry among non-diabetic ESRD cases in comparison to controls without nephropathy. Moreover, in this study, one or more susceptibility alleles for non-diabetic ESRD have been proposed within *MYH9* gene in African American. However, for the time being it is not known which genetic variant is responsible for this susceptibility, since none of the SNPs typed in this study influence gene transcription or translation directly. MYH9 encodes for the protein non-muscle myosin heavy chain (class II, isoform type A). According to authors, aggregation of abnormal myosin and podocyte and tubular cells cytoskeleton injury could result in progressive kidney disease. Nevertheless, they did not reveal how sequence variations in MYH9 directly resulted in development and progression of non-diabetic kidney disease. The identification of MYH9 gene suggests a novel pathway of kidney disease progression. The determination of causal variants and unravelling the pathophysiologic mechanisms will help to design new treatment strategies decreasing the risk of progression to ESRD among African Americans or hampering the course of disease [65]. However, more recent studies suggest that the presence of two risk alleles (termed G1 and G2) in the last exon of gene encoding apolipoprotein-L1 (APOL1), not variations within MYH9, are associated with 5–29 times higher odds of severe kidney disease, including non-diabetic ESRD, hypertension-related ESRD, focal segmental glomerulosclerosis, and HIV-related nephropathy [66,67,68,69]. In the large community-based study of 15,792 adults recruited from four US communities (sub-analysis of ARIC Study), participants carrying two risk alleles were at an increased risk of developing incident CKD as well as ESRD events in comparison to patients lacking these alleles or having only one of them [70]. Moreover, they reported that over 31% of African American CKD carriers of two APOL1 risk alleles progressed to ESRD, while only 13% of patients with CKD and with zero or one risk allele faced such aggravation of renal function during over 6 years of follow-up. Quite high prevalence of G1 and G2 risk alleles among African Americans in comparison to Americans with European ancestry could be one of reasons for the increased kidney disease burden in African Americans. In the Dallas Heart Study including 1776 African Americans, carriers of two *APOL1* risk alleles had three- to four-fold increased odds of prevalent microalbuminuria and reduced estimated GFR (eGFR; eGFR < 60 mL/min per 1.73 m^2^) in comparison with participants with zero or one risk allele. This relationship was influenced by diabetes status [70]. 

Methylenetetrahydrofolate reductase (MTHFR) is another factor influencing progression to ESRD. The single nucleotide polymorphism 677 C/T in the gene encoding MTHFR, which is associated with amino acid exchange (Ala/Val) resulting in reduced enzymatic activity, was demonstrated to be more frequent (T and TT genotype) in patients with diabetic nephropathy than in those without (*p* < 0.05) [71]. Moreover, it seems that this SNP influences the progression of renal failure to end-stage renal disease (ESRD) in dialyzed patients with diabetic nephropathy. According to Ksiazek et al. [71] study, mean time from diagnosis to the onset of ESRD was 3.6 years for patients with the TT genotype compared with 7.3 years for the CC genotype (*p* < 0.01). Authors suggested that the C677T mutation in the MTHFR gene predisposes patients with type 2 diabetes to develop diabetic nephropathy. 

Some studies suggest that renal myofibroblast infiltration strongly correlates with renal function decline in several chronic renal diseases [72]. The study of 38 biopsy samples of renal tissue from renal transplant patients demonstrated a considerable correlation between interstitial smooth muscle actin α (α-SMA) expression in time-zero biopsies and annual loss in glomerular filtration rate (GFR) during the post-transplantation course (*r* = 0.60, *p* < 0.001). In patients with progressive reduction of GFR, increased expression of α-SMA and enhanced interstitial fibrosis was observed in comparison to non-progressors. Authors concluded that the determination of α-SMA expression and the presence of interstitial fibrosis can strongly predict chronic renal allograft dysfunctions [72]. 

l-Fatty acid binding protein (l-FABP) has been suggested to be a clinical marker for monitoring of CKD [73]. l-FABP binds fatty acids and transports them to mitochondria or peroxisomes, where the fatty acids are β-oxidized [74,75,76]. Due to the fact that l-FABP has a high affinity for long-chain fatty acid oxidation products and a capacity to bind them, it can be an effective endogenous antioxidant [77]. Multicentre trial involving non-diabetic CKD patients followed for a year demonstrated that urinary l-FABP level was considerably higher in with progressive disease in comparison to stable CKD. Urinary l-FABP was also shown to be more sensitive than urinary protein in predicting the progression of CKD (93.8% and 68.8%, respectively) but less specific than it (62.5% and 93.8% respectively). Changes in urinary l-FABP correlated with the progression of CKD in time (*r* = −0.32, *p* < 0.05). Due to the fact that urinary excretion of l-FABP increases with the deterioration of kidney function, it can be utilized as a useful clinical marker in the monitoring of CKD [73]. 

Some biological substances may also protect against ESRD. Vitamin D is a fat-soluble steroid hormone which regulate a variety of biological processes, including transcription of several genes involved in CKD disease mechanisms [78]. Study of 222 subjects with and without ESRD demonstrated that G allele of the Vitamin D Receptor (VDR) gene BsmI polymorphism was associated with protection against ESRD [78]. GG genotype was 2.5 times more frequent in the group without ESRD. In this study, allele G was associated with protection against ESRD: groups without versus with ESRD (GG) × (GA + AA): OR = 2.5, 95% CI = 1.4–4.6, *p* = 0.00; (G × A): OR = 1.5, 95% CI = 1.0–2.3, *p* = 0.02; (TG + CG) × (TA + CA): OR = 1.5, 95% CI = 1.0–2.3, *p* = 0.02. These findings have to be validated in large studies. 

## 4. Treatment of Renal Diseases

Drugs pharmacokinetics and pharmacodynamics determine their therapeutic and toxicological effects. Allelic variants in genes coding proteins responsible for influencing drug transport, metabolism, and mechanism of action may be responsible for altered drug response and toxicity [79]. Carriers of alleles associated reduced drug metabolism have higher plasma drug concentrations than wild-type homozygous individuals, which may be associated with toxic effects. On the other hand, persons with alleles connected with increased rate of drug metabolism may require higher drug doses to fully benefit from its effects. There are several types of drugs used in the treatment of renal disorders that are characterized by a high variability in pharmacokinetic behaviour and by a poor correlation between drug concentrations and pharmocodynamic effects [80,81,82,83]. Therefore there is a need to “tailor” the dose of these drugs to avoid toxicity and to obtain full efficacy.

Azathioprine (AZA) which is a purine anti-metabolite used widely in nephrology has been demonstrated to be highly polymorphic. Polymorphisms within gene encoding cytosolic enzyme thiopurine S-methyltransferase (TPMT gene) have an important impact on its [84,85]. About 20 polymorphisms within this gene (TPMT*2–*18) result in intermediate or very low activity (in homozygous carriers of mutation) in comparison to TPMT*1 wild-type allele [86]. Reduction or loss of ability to metabolize AZA are associated high blood levels and greater risk of severe and potentially life-threatening myelotoxicity [87]. However, elevated TPMT activity was shown to be associated with higher risk of acute rejection [88]. Therefore, it seems that TPMT genotyping before the start of AZA treatment may help to lower the risk of complications. 

Genetic variability is also important in the case of pharmaceutical treatment. Various severe glomerular diseases (e.g., lupus nephritis) are treated with cyclophosphamide (CYC) which is converted in human body to its active metabolite—phosphoramide mustard. Cytochrome P450 enzyme system (especially CYP2B6 and CYP2C19 enzymes) plays an important role in this transformation. However, there are numerous polymorphisms within their genes, which are associated with the formation of inactive enzymes or enzymes with reduced activity. CYP2B6 allele 5 (CYP2B6*5) in which cysteine replaces the arginine at position 487 and CYP2C19 allele 2 (CYP2C19*2) are the most frequent variants [89,90]. Takada et al. [91] study confirmed the hypothesis that homozygous patients for CYP2B6*5 or CYP2C19*2 were poor metabolizers of CYC, they less frequently achieved complete remission and therefore they were at greater risk of progression to end-stage kidney disease in comparison to heterozygotic carriers. Also the pharmacokinetics of mTOR (mechanistic target of rapamycin, mammalian target of rapamycin) inhibitors, which are used as anti-cancer and immunosuppressive agents (sirolimus, everolimus) was shown to be influenced by polymorphisms in CYP3A4/CYP3A5 genes. Le Meur et al. [92] study demonstrated that sirolimus metabolic activity and oral clearance were significantly decreased in patients who are homozygous for the CYP3A5*3 single-nucleotide polymorphism. They showed that patients with the CYP3A5*1/*1 and *1/*3 genotypes required a significantly higher sirolimus daily dose to achieve the same blood concentration at steady state as *3/*3 patients. It seems that the determination of this polymorphism could be useful in the optimization of sirolimus management in transplant recipients. Sirolimus concentration can be also determined in blood. Due to the long half-life of sirolimus, the adjustment of dose should be performed on the basis of blood levels obtained more than 5–7 days after initiation of therapy or dosage change [93]. Moreover, sirolimus concentration monitoring ought to be carried out every week during the first month and every 2 weeks for the second month. After the first 2 months, sirolimus monitoring is not necessary in all patients, but may be required in order to achieve target concentrations in certain populations of patients [93]. The analysis of polymorphisms within CYP3A5 genes may facilitate initial adjustment of dose and indicate patients who need higher sirolimus doses. 

Problems with variations in cytochrome P450 genotype are also associated with tacrolimus (Tac) treatment [94]. This drug is effective in the prevention of acute rejection, however, some studies suggest its toxicity due to inter-individual variability in pharmacokinetics and pharmacodynamics. According to studies, CYP3A4*22, CYP3A4*26, and POR*28 are associated with Tac dose requirements and may influence the effects of Tac therapy. Other studies have demonstrated that a SNP- CYP3A5*3 (SNP rs776746) is the main regulator of the optimum dose [95,96,97]. This variant within intron 3 of the CYP3A5 gene influences pre-mRNA processing resulting in disturbed splicing between exons 3 and 4 and in consequence the resulting abnormal, unstable mRNA is eliminated by the cell and therefore it fails to synthesise protein [98,99]. Homozygous carriers of CYP3A5*3 allele do not have this protein, while in heterozygous carriers the level of the protein is reduced. The analysis of CYP3A5*3 alleles enables the identification of patients who are “slow metabolisers” (homozygous CYP3A5*3*3), “intermediate metabolisers” or “fast metabolisers” (heterozygous CYP3A5*1*3 and homozygous CYP3A5*1*1, respectively). The latter type of patients requires higher doses of tacrolimus in order to achieve target levels of tacrolimus [97,100,101]. Also other gene variants might influence Tac metabolism. Numerous studies indicated significant differences in CYP3A5 allele frequencies between ethnic groups showing that about 80% of Caucasians are slow metabolisers while most black patients are homozygous CYP3A5 *1*1 (fast metabolisers) [102,103]. Tacrolimus has also been shown to be metabolized by P450-3A4. Polymorphisms within CYP3A4 gene affects cytochrome activity and in consequence basal drug levels/doses [97]. Polymorphism CYP3A4*1B within promoter region was shown to be associated with higher protein levels and requirement for greater drug doses [104,105]. A study focused at the determination of advantages associated with tacrolimus initial dose adjustment according to genotype (slow- and fast-metabolizers) in comparison to the current method of dose choice, demonstrated that after three days of treatment the percentage of patients achieving target value C0 was higher in group with dose adjustment (43.2% vs. 29.1%, *p* = 0.03) and these patients required fewer dose modifications [106]. However, no significant differences were observed in frequency of acute rejection or in renal function values.

Some reports suggest that glucocorticoids treatment may be associated with toxicity, while other demonstrate that some patients are resistant to glucocorticoids due to overexpression of glucocorticoid the receptor [107,108]. Miura et al. [109] have shown that nuclear receptor subfamily 1, group I, member 2 (NR1I2, A7635G) allelic variants, influenced patient variability of plasma prednisolone concentrations in renal transplant recipients on maintenance immunosuppressive treatment. Patients carrying the NR1I27635G allele had higher metabolic activity for prednisolone in the intestine, greatly reducing its maximal plasma concentration. The issue of pharmacogenomics of immunosuppressive drugs is very important for kidney transplant patients. 

On the basis of DNA microarrays, also the determination of molecular variations suggesting the existence of distinct molecular and prognostic variants of acute rejection is possible. Differences in gene-expression patterns in samples from patients with acute rejection are frequently associated with alterations in the composition and activation of infiltrating lymphocytes [110]. According to studies, despite similar clinical and histologic phenotypes, three molecular groups can be identified in children with acute allograft. Total renal biopsy RNA expression (microarray assay) demonstrated enhanced expression of B cell genes, while immunohistochemistry revealed the abundance of B cells in renal biopsy tissue in patients from one of these three groups [110]. CD20+ lymphoid aggregates were found to be associated with poorer graft outcomes. It seems that in patients with such an infiltration, early treatment with a monoclonal antibody against CD20 (rituximab) may be beneficial [111]. Zarkhin et al. found that the identification of molecular subtype of acute rejection before treatment may be used to indicate patients who are likely to be steroid resistant and who may benefit from a different antirejection protocol [112]. 

## 5. Risk Factors in Kidney Diseases and Prognosis

Chronic kidney disease (CKD) is a serious condition, which untreated lead to end-stage renal disease, necessity of dialysis or kidney transplantation, and premature mortality. However, it affects also other systems resulting in hypertension and cardiovascular disease [23]. In patients with chronic kidney disease, vascular calcification (VC) is an important risk factor which leads to increased arterial stiffness, cardiovascular disease (CVD) and finally to mortality in this group of patients. Low serum levels of fetuin A, which mediates the inhibition of calcium-phosphate precipitation and VC, has been shown to be associated with poorer survival of hemodialysis patients. Until now, four SNPs in fetuin A gene have been quite well studied—Thr248Met (C/T), Thr256Ser (C/G), Asp276Asn (G/A) and Arg317Cys (C/T). Stenvinkel et al. [113] study demonstrated reduced serum levels of fetuin (*p* < 0.0001) and higher all-cause (log-rank 34.2; *p* < 0.0001) and CVD mortality (log-rank 23.4; *p* < 0.0001) (related to concurrent inflammation) in CKD patients who were carriers of Ser256 allele. 

Due to the fact that systemic inflammation is considered to be a factor promoting the development of vascular disease and protein energy wasting (PEW), polymorphisms within genes encoding inflammatory cytokines and their receptors may be associated with worse prognosis and higher mortality of CKD patients. In the presence of inflammatory state, CCR5 receptor contributes to atherogenesis via binding of its ligands, which in turn mediates the recruitment of inflammatory cells to the endothelium. Insertion/deletion (I/D) (32-bp deletion in the open reading frame) polymorphism within the gene encoding CC chemokine receptor 5 (CCR5) has been shown to results in the formation of dysfunctional protein due to premature termination of the protein and its sequestration in endoplasmic reticulum [114,115]. DD genotype was suggested to exert beneficial impact on CVD risk. Multicenter, prospective NEtherlands COoperative Study on the Adequacy of Dialysis (NECOSAD) cohort confirmed that CCR5 Δ32 polymorphism attenuated the adverse effects of inflammation on overall and cardiovascular mortality in ESRD [115]. In this study, patients with hsCRP > 10 mg/L and insertion genotype had an increased all-cause mortality risk compared with persons with the same genotype but hsCRP ≤ 10 mg/L (HR: 1.82; 95% CI: 1.29 to 2.58). Carriers of deletion allele even with hsCRP > 10 mg/L, demonstrated significantly lower HR for all-cause mortality, and even more pronounced diminished cardiovascular mortality. Increased mortality of inflamed patients with I/I genotype in comparison to those with D/D genotype suggests that CCR5 genotype may mitigate adverse effects of inflammation on overall and cardiovascular CKD mortality. It seems that the blockade of CCR5 might be utilized in the future as a novel therapeutic approach improving cardiovascular condition by interfering with systemic inflammation. Preliminary study on animal model revealed reduced progression of atherosclerosis in hypercholesterolemic mice treated with CCR5 antagonist Met-RANTES [116]. 

Also, SNPs within another proinflammatory cytokines predict cardiovascular disease and mortality in the uremic milieu. Polymorphism within the promoter of interleukin-6 (IL-6) gene (G allele in 174 G/C SNP) has been associated with increased comorbidity [117]. Another study indicated that the influence of this polymorphisms depended on the presence of 162Val allele [118]. Tumor necrosis factor-α (TNF-α) influences vascular calcification through the promotion of osteoblastic differentiation. There are many polymorphisms within TNF-α In HD patients, 308 A allele (308G/A) is associated with increased TNF-α production, reduced levels of S-albumin, enhanced comorbidity and diminished functionality [117]. TNF-β is a proinflammatory cytokine which stimulates the production of several other proinflammatory factors, including IL-6 and P-selectin [119]. Thr36Asn SNP within lymphotoxin-a (LTA) gene encoding by TNF-β was associated with greater risk of CVD and CVD mortality in dialysis patients. Zaza et al. [120] analysed the association between the activation of specific transcriptome and the chronic inflammatory state in HD and PD patients. They found an independent relationship between macrophage migration inhibitory factor (MIF), interleukin 8 receptor b (IL8RB), and chemokine (C-X-C motif) ligand 12 (CXCL12) and with inflammation. CXCL12 and IL8RB were shown to be inversely correlated to CRP levels and highly expressed in CKD patients, while, MIF was increased in HD patients and directly correlated with CRP. It has been suggested that these genes may be used as potential targets for future therapeutic approaches to decrease inflammation in dialysis patients [120].

Finally, in patients with renal and vascular disease, the renin-angiotensin system (RAS) up-regulation has been reported [121]. Insertion/deletion (I/D) polymorphism within intron 16 of angiotensin I converting enzyme (ACE) gene is associated with have higher ACE levels in DD carriers. Increased levels of ACE has been shown to correlate with not only left-ventricular hypertrophy (LVH) and higher carotid intima-media thickness in ESRD [121], but also with the rate of renal function decline (faster in D allele carriers) [122]. Moreover, Perez-Oller et al. [123] suggested that DD homozygotes were more likely to develop CKD at a younger age and to have a worse prognosis. Also polymorphism within gene encoding angiotensinogen (AGT) (Met235Thr) is associated with high AGT levels, increased risk of concentric left ventricle hypertrophy and vascular complications in renal populations [124].

**Figure 1:**
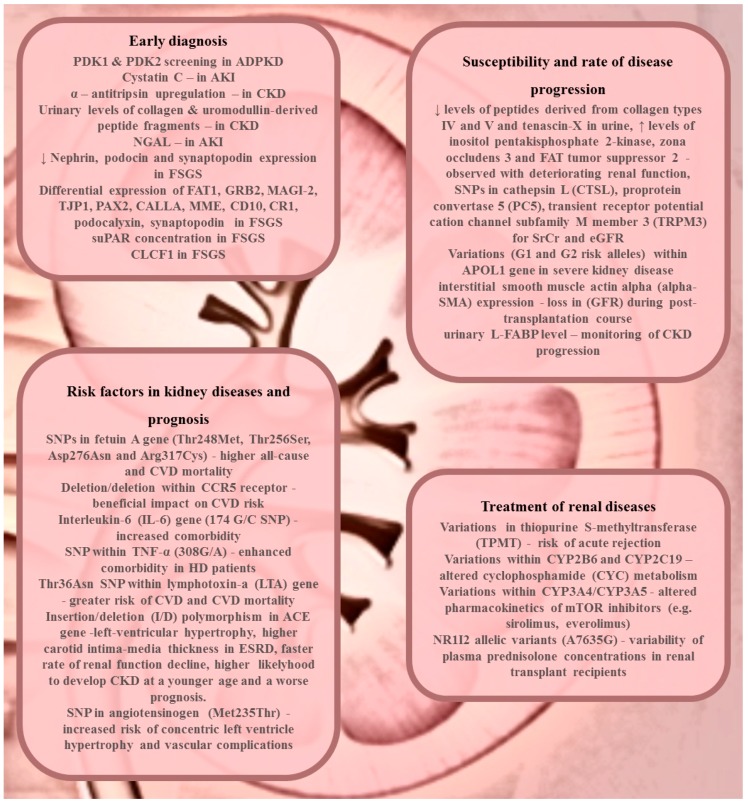
CKD-related biomarkers in personalized medicine—summary

All described biomarkers are presented in Figure 1.

## 6. Financial Aspect

CKD and end-stage renal disease (ESRD) are associated not only with the suffering and poor quality of life for those afflicted but also with very high costs of treatment incurred by the country. CKD is also responsible for premature death and high economic price from both the private and public sectors. CKD and ESRD are very expensive to treat [125]. In England, annual spending of National Health Service (NHS) on kidney care was estimated at £445 million in 2002 [126]. Programme budgeting analysis estimated the total NHS expenditure on kidney care, including CKD, at £1.64 billion in years 2009–2010 [127]. In turn, in United States annual Medicare expenses (per person) attributable to CKD were $1700 for stage 2, $3500 for stage 3, and $12,700 for stage 4, adjusted to 2010 dollars [128]. According to USRDS report of 2010, Medicare spent in 2009 $29 billion for people with ESRD, which was almost 6% of the annual Medicare budget [129]. The annual expenses incurred by Medicare in 2011 on adults aged 65 years or older with all stages of CKD were $20,432 per person [130]. The results of Honeycutt et al. [128] analysis show that medical costs attributable to CKD are substantial even during the early stages and they increase as disease severity worsens. Moreover, this study underlines the need to diagnose CKD in its earliest stages to prevent disease progression and avoid high medical costs attributable to the latter stages of the disease. The prevention of CKD development, including diabetes and hypertension control, may be associated with significant medical cost savings. The identification of people with CKD in its early stages may also help to reduce costs associated with the treatment of kidney-related comorbidities including cardiovascular disease [131]. The use of personalized medicine in case of patients with early stages of CKD without microalbuminuria may seem unjustified from economical point of view, however, the identification of biomarkers which would indicate patients at high risk of disease progression may lead to the reduction of future expenses. Despite the fact that there are no specific treatment of patients with early stages of CKD (only measures aiming at the slowing down of disease progression) their identification is important. A cost analysis is required to confirm that the costs of biomarkers analysis at an early population level will be more favourable than the extensive costs associated with the treatment of ESRD patients or transplantation.

## 7. Conclusions

Studies indicate that renal diseases are influenced not only by environmental but also by genetic factors. Therefore, it seems that the analysis of genetic aspects should be a natural component of clinical and experimental studies. Genetic findings should be supplemented with the analysis of the related protein, resulting in the combined genomic, proteomic and metabolomic approach. According to Padullés et al. [3] personalization of nephrological therapy can be achieved by using pharmacogenetic/genomic/dynamic approach or a biomarker approach. There are available results of small studies, in which pharmacogenetics, pharmacogenomics and pharmacodynamics have been utilized in some types of kidney disease, however, their results are not conclusive. For the time being there are no established biomarkers which could be used to tailor therapies.

Genetic studies should always be confirmed in larger populations with preferably well-controlled ethnicity. Whole-genome examinations may revolutionize the approach to the disease and the patient resulting in the creation of “personalized medicine” with new diagnostic and treatment strategies designed on the basis of genetic background of each individual. The ethnic background is important due to the fact that it may influence susceptibility to diseases. Considerable variation in disease prevalence was observed between different ethnic groups. For example, individuals of African origin, both in Africa and when migrated to the Caribbean or to Europe, have a much lower prevalence of CHD [132], but in the same time Blacks and African Americans suffer from kidney failure at a significantly higher rate than Caucasians [133]. Risk stratification performed in a large population is further translated by means of personalized medicine into individual approach to a single patient. The identification of high-risk patients in pharmacogenomics analyses will help to avoid many unwarranted side effects while optimizing treatment efficacy for individual patients. However, most (all) the aforementioned results should be confirmed in large studies. Moreover, it seems that in complex diseases, single biomarker will be not sufficient to model a specific aspect of the disease and that biomarker panels will be required [3].
